# Exploring cross-modal perception in a virtual classroom: the effect of visual stimuli on auditory selective attention

**DOI:** 10.3389/fpsyg.2025.1512851

**Published:** 2025-11-14

**Authors:** Carolin Breuer, Lukas Jonathan Vollmer, Larissa Leist, Stephan Fremerey, Alexander Raake, Maria Klatte, Janina Fels

**Affiliations:** 1 Institute for Hearing Technology and Acoustics, RWTH Aachen University, Aachen, Germany; 2 Cognitive and Developmental Psychology, RPTU Kaiserslautern-Landau, Kaiserslautern, Germany; 3 Biological Psychology, RPTU Kaiserslautern-Landau, Kaiserslautern, Germany; 4 Audiovisual Technology Group, Technische Universität Ilmenau, Ilmenau, Germany; 5 Institute for Communications Engineering, RWTH Aachen University, Aachen, Germany

**Keywords:** audio-visual attention, auditory selective attention, binaural hearing, virtual reality, visual priming, attention switching

## Abstract

In virtual reality research, distinguishing between auditory and visual influences on perception has become increasingly challenging. To study auditory selective attention in more close-to-real-life settings, an auditory task was adapted to a virtual classroom. The new environment suggested evidence of increased attention, possibly introduced by the visual representation, gamification effects, and immersion. This could engage participants more effectively. To delve deeper into the impact of cross-modal effects, the paradigm was extended by visual stimuli. Participants were initially tasked with directing their auditory attention to a cued spatial position and categorizing animal names played from that position while ignoring distracting sounds. Animal pictures introduced in Experiment 1 were either congruent or incongruent with the auditory target stimuli, thus either supporting or competing with the auditory information. The concurrent presentation of animal pictures with the animal names increased response times compared to the auditory condition, and incongruent visual stimuli increased response times more than congruent ones. Fewer errors were made with congruent compared to incongruent pictures, and error rates of the auditory condition fell in between. When the visual stimulus was presented 750 ms or 500 ms before the auditory stimuli in Experiment 2, auditory and visual congruence effects interacted. In the 500 ms case, visually congruent stimuli decreased error rates in auditory incongruent trials. Conversely, visually incongruent stimuli decreased error rates on auditory incongruent trials at 750 ms. This reversal of effects suggests a positive priming effect at 500 ms and a semantic inhibition of return effect at 750 ms. Taken together, these findings indicate that cross-modal priming is at least partially different from multisensory integration.

## 1 Introduction

Although the term auditory selective attention (ASA) switching might be unfamiliar, this mechanism is employed frequently in everyday life. A common example is having a conversation in a crowded room, where one needs to listen to the conversational partner while filtering noise sources such as other conversations or music. The ability to focus on one specific sound source (the conversational partner) is called auditory selective attention, and the reorientation, e.g., to listen to a different person, is called attention switching (Cherry, 1953; Koch et al., 2011). Switching auditory selective attention is the key mechanism that enables successful human communication in dynamic and challenging acoustic environments. Laboratory experiments investigating the working principles of ASA and its switching process often fail to account for real-world influences such as auditory spatial information and cross-modal audio-visual effects. However, how well findings from such controlled settings generalize to real-life complexity remains unclear. Particularly in educational settings, complex and dynamic scenarios may divert attention away from learning materials.

Motivated by the works of Doyle (1973); Jones et al. (2015); Peng et al. (2018), which indicated developmental effects in auditory selective attention, as well as a higher noise sensitivity in children than adults (Loh et al., 2022), the present research aims to create a more realistic audio-visual task to investigate real-world influences on ASA, while remaining suitable for children. In other words, this study seeks to bridge the gap between controlled ASA paradigms and the multisensory, distraction-rich environments people actually encounter. In real-world classroom settings, auditory and visual information are inherently intertwined, even when only auditory attention is of interest. Such environments typically include both visual and acoustic targets and distractors, for example, a teacher writing on the board or displaying images to support spoken content, alongside background conversations among students. Additionally, students themselves may take notes or create drawings to aid their learning process. Speech, moreover, is frequently accompanied by visual cues such as lip movements and gestures, which enhance comprehension of the auditory message (see, e.g., Peelle and Sommers, 2015).

This interaction between multiple sensory modalities, such as auditory and visual stimuli, is commonly referred to as multisensory integration (Stein and Stanford, 2008; Spence, 2007). In this context, one important phenomenon is multisensory enhancement, where the presence of a stimulus in one modality enhances perception in another (Stein and Stanford, 2008). Numerous studies have shown that even task-irrelevant visual stimuli can facilitate auditory perception, for example, by improving spatial localization, loudness perception, or frequency discrimination. A comprehensive overview of these effects is provided by Opoku-Baah et al. (2021). However, such effects are rarely examined in scenarios that combine realistic environments with experimental control.

To understand real-world, cross-modal effects, many researchers create virtual reality (VR) scenarios, which balance real-world likeness and experimental control. Although there is a clear trend toward using the technology for psychological research (Martin et al., 2022), the impact of the virtual environment and the usually head-worn hardware on cognition is not yet fully understood. This uncertainty makes VR an interesting research platform for exploring ASA in settings that are both controlled and realistic.

Building upon the voluntary auditory selective attention switching task by Koch et al. (2011); Fels et al. (2016); Loh et al. (2022), an audio-visual VR classroom scenario was created (Breuer et al., 2022). In this task, participants need to classify whether a target animal name belongs to the category of flying or non-flying animals. Here, the auditory stimuli are played back from one of four spatial positions around the participant (front, back, left, right). To enforce auditory spatial attention, a distracting animal name is played simultaneously from a different position. Thus, participants need to focus on the cued target position and decide on the target category. This task required not only spatial detection but also semantic processing of the stimuli.

Comparing performance in the audio-only and VR versions of the ASA paradigm, similar overall patterns were observed. However, adult participants made significantly fewer errors and responded faster in the VR setting. This exploratory study, therefore, suggested increased attentional focus or engagement in VR compared to an audio-only study (Breuer et al., 2022; Loh et al., 2022). These results raise the question of whether the observed VR benefit stems from the immersive environment itself or from visual cues supporting auditory attention.

Other studies support the idea that VR alters task performance, but the direction and interpretation of these effects vary. Makransky et al. (2019), for instance, found a higher presence and cognitive load in VR compared to a desktop display, yet participants reported learning less, possibly due to overstimulation. Similarly, Albus and Seufert (2023) argue that the VR environment might induce excessive visual load and thus distracts from the actual task. In contrast, other studies found enhanced motivation or task performance in VR settings (Wan et al., 2021; Li et al., 2020; Redlinger et al., 2021; Wenk et al., 2023), which could be related to visualization, gamification, or the hardware.

Based on these mixed findings, Experiment 1 of the present research aimed at investigating whether visualization effects might have enhanced the attentional focus in the VR study by Breuer et al. (2022). The virtual environment was therefore extended with visual stimuli related to the attention-switching task, while keeping the closer-to-real-life design. However, the virtual environment itself was not the focus of investigation here, as it was adopted without modification from Breuer et al. (2022). In that work, established measures such as the Simulator Sickness Questionnaire (Kennedy et al., 1993) and the Igroup Presence Questionnaire (Schubert, 2003) appeared insufficiently sensitive in the context of this attention task and participant group. Therefore, in the present study, the VR environment was used primarily to provide a plausible and engaging task context rather than as a variable of interest. This design ensured that any observed differences could be attributed to the manipulation of task-related visual information rather than changes in the VR setting itself.

As stated, audio-visual enhancement is well investigated in classical laboratory environments (Stein and Stanford, 2008). For example, attending to sounds can enhance the detection of visual stimuli, when auditory stimuli are played back simultaneously with visual ones (McDonald et al., 2000; Spence, 2013). However, this spatial rule applies primarily for congruent sounds, not words (Marian et al., 2021).

Instead, the present study specifically investigates cross-modal semantic priming, which refers to the facilitation of the semantic processing of stimuli, when a related stimulus is presented, e.g., seeing an image of a *cat* facilitates the semantic processing when hearing the word *dog* (Chng et al., 2019). Similarly, Downing et al. (2015) found lower response times in detecting target objects in audio-visual than audio- or visual-only trials when showing the image of an animal together with the respective sound, e.g., the image of a cat with *meow*. Interestingly, the audio-visual speed-up appears to encompass a multisensory redundancy component (i.e., content independent unimodal vs. multimodal stimulation), and a semantic component (Roberts et al., 2024). Regarding the semantic component, Barutchu et al. (2013) suggest that multisensory facilitation appears only when both components of the audio-visual stimuli are targets, i.e., when auditory and visual parts of the stimuli hold relevant information for the participants. On the other hand, Molholm et al. (2007) conclude that attending to an object within one sensory modality results in coactivation of that object's representations in ignored sensory modalities.

A detailed review on multisensory integration focusing on semantic cross-modal processing is given by Wegner-Clemens et al. (2024). They further give plenty of examples where semantic processing of task-irrelevant auditory stimuli guided visual spatial attention (McDonald et al., 2000; Nah et al., 2021; Peacock et al., 2019; Iordanescu et al., 2010). However, Wegner-Clemens et al. (2024) underline the need for real-life studies, since these scenarios incorporate a variety of task-unrelated stimuli from different modalities. Similarly, the current study is situated in a virtual classroom environment, which offers visual distraction, e.g., in the form of furniture, and engages the participant through a game-like interaction. Therefore, such a complex environment in itself requires a different attentional focus than a laboratory setting. Thus, the question arises whether the same semantic priming effects hold in such a complex VR scenario.

While Experiment 1 of the present study was concerned with the general semantic processing of task-irrelevant visual stimuli on the voluntary switching of auditory selective attention, Experiment 2 investigated whether this effect is strengthened by priming. This question is particularly relevant since, in real-world settings, sensory cues rarely coincide. To explore this, the duration of the visual stimulus was extended to assess whether prolonged exposure enhances cross-modal integration and influences auditory attention and decision-making.

In previous investigations on cross-modal priming, Holcomb and Anderson (1993) used a lexical decision task to explore semantic priming effects of written prime words on auditory target words (and vice versa), which were either semantically related or unrelated. They found a semantic priming effect, i.e., related vs. unrelated words, that tended to be larger with increasing stimulus onset asynchrony (SOA) between visual prime and auditory target. In a similar study, Kircher et al. (2009) used pictures and written words to prime written target words with a fixed onset asynchrony of 350 ms. They, too, found a semantic priming benefit for related over unrelated prime-target pairs but no difference between picture and word primes. Later, McQueen and Huettig (2014) showed semantic priming effects for written words and line drawings on auditory target words using priming intervals of 1,000 ms and 2,400 ms, which were similar in magnitude. These results suggest that the type of visual display (written word or picture) does not affect priming in lexical decision tasks. Nonetheless, these effects have not been investigated in a VR environment so far.

Previous studies did not examine priming under conditions requiring selective attention or attention switching. However, context effects, such as priming, can improve or even interfere with task performance. While Shigeno (2017) shows the benefit of related primes on target word identification in adverse listening situations, de Groot et al. (2017) demonstrate bias effects toward a memorized prime word in a visual search task. In task-switching, stimuli unrelated to the to-be-performed tasks were shown to improve performance when both the task and the response were repeated (Benini et al., 2023). However, including the irrelevant visual stimulus in the task was only beneficial when it occurred together with the cue. Thus, unrelated and related but task-irrelevant stimuli can affect task performance, although the extent of the effect may depend on task demands and the primary processing modality (Schoepper and Frings, 2023).

The present study addressed three main questions: (1) whether the presented information (animal names and pictures) is processed cross-modally in a virtual classroom; (2) whether visual stimuli facilitate auditory attention and classification; and (3) whether prolonged visual presentation enhances cross-modal integration. These questions aim to clarify whether established priming and multisensory integration effects extend to complex, immersive VR contexts.

## 2 Experiment 1: effects of semantic priming on auditory selective attention

### 2.1 Methods

#### 2.1.1 Participants

Although the virtual scenario was originally designed for children, this study was conducted with adults to establish a baseline for future developmental research. The scenario was also appropriate for young adults, as it reflected familiar experiences such as seminars or lectures, making it contextually meaningful.

To determine the number of participants, a power analysis was performed in G*Power (Faul et al., 2007) assuming an effect size of *f* = 0.25, an α error probability of α = 0.05, and a power of 1−β = 0.95. With 15 repetitions per trial, a sample size of 13 participants was calculated. To increase robustness, a subject group of 24 participants was chosen. This is in line with previous studies using the paradigm (Koch et al., 2011; Fels et al., 2016; Oberem et al., 2014; Loh et al., 2022; Breuer et al., 2022). In Experiment 1, 24 adults participated (age: 20–27 years, M = 23.46 years, SD = 1.70 years, 13 female). All participants were fluent German speakers with normal hearing (within 25 dB (HL), World Health Organization, 1991) measured by an Auritec Ear 3.0 audiometer using an ascending pure tone audiometry between 250 Hz and 8 KHz (AURITEC, 2024). Further, normal or corrected to normal vision acuity (20/30) measured by a Snellen chart (Snellen, 1862) was required. Using a subset of Ishihara color charts (charts: 1, 2, 4, 8, 10, 14, according to instructions for quick testing), normal color vision was tested (Ishihara, 2009). None of the participants had participated in a listening experiment on auditory selective attention within the last six months prior to the study. Informed consent was obtained from all participants before the participant screening and before the study. The study was performed in accordance with the Declaration of Helsinki. A statement of non-objection was obtained from the Medical Ethics Committee at RWTH Aachen University with the protocol number EK 395-19.

#### 2.1.2 Experimental task and design

The experimental task was to classify whether a previously cued animal name belonged to a flying animal or not. Two auditory stimuli were played back from one of four possible positions around the participant (front, back, left, right). In the virtual classroom scenario, the distance between the audio source and the participant was 2 m. In every trial, two animal names were played back simultaneously, but from different positions. An auditory cue, played back from one of the positions 500 ms before the animal names, marked the target position. The other distracting stimulus needed to be ignored. The target position could either be repeated or switched between consecutive trials, while the distractor position was changed each trial.

To mimic classroom behavior, where a teacher writes on the board, a visual stimulus was displayed on the virtual blackboard in front of the participant. Because previous studies (Pérez et al., 2020; McQueen and Huettig, 2014) found no differences between pictures and written words in auditory word recognition, images of the animal names were used as visual stimuli. This will allow for the testing of young children who are not yet literate in future studies. The visual stimulus was visible in half of the trials and was presented simultaneously to the auditory stimuli. In both experiments, each trial, i.e., each possible combination of conditions, was repeated 15 times.

During the experiment, the spatial and temporal representation, as well as the content of the auditory and visual stimuli, were manipulated. For simplicity, the cross-modal spatial component was omitted, and the visual stimulus was only presented in front of the participant.

The independent variables are described in detail in the following section. To represent the task performance, response times (RT in ms from target stimulus onset) and error rates (ER in %) were measured.

##### 2.1.2.1 Attention transition

**Figure 1:**
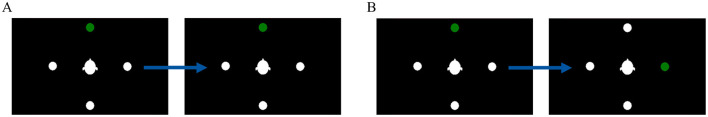
Attention transition (AT). **(A)** Attention repetition. **(B)** Attention switch. As adapted from Breuer et al. (2022).

At the beginning of each trial, the cue marked one of four possible positions as the target position. This position could either be repeated (e.g., *front-front*) or switched (e.g., *front-right*) between trials (see Figure 1). This switching of the target position requires a refocusing of the auditory attention. This variable is called *attention transition* (AT) and has two levels (*repetition* and *switch*).

##### 2.1.2.2 Target-distractor position-combination

**Figure 2:**
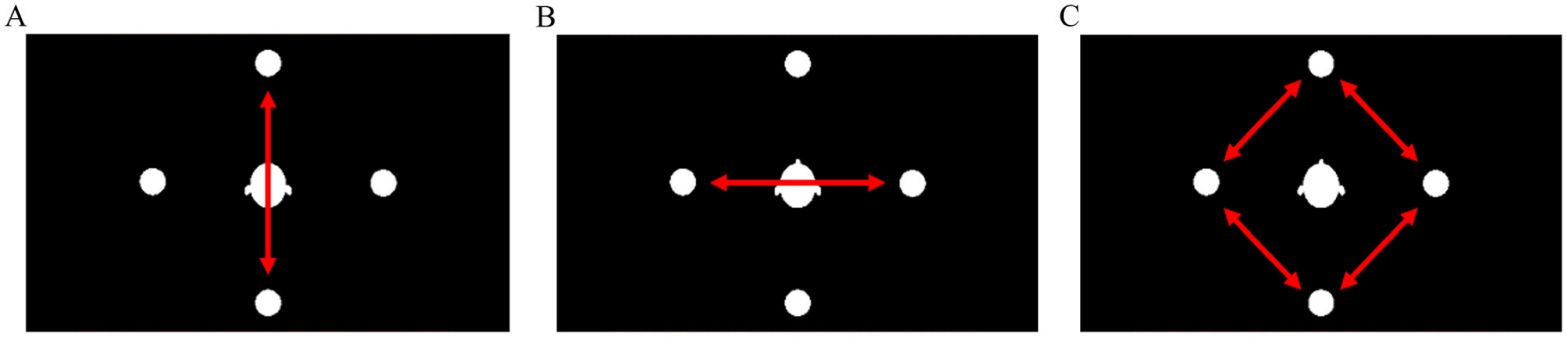
Target-distractor position-combination (TD-PC). **(A)** Front-back. **(B)** Left-right. **(C)** Next-to. As adapted from Breuer et al. (2022).

The combination of target and distractor position was varied between trials, since the distractor position was changed in each trial. The *target-distractor position-combination* (TD-PC) as illustrated in Figure 2 describes the spatial relation between the target and distractor stimuli. It had three levels and could either be *left-right, front-back*, or *next-to* (e.g., *left-front*).

##### 2.1.2.3 Auditory congruence

During each trial, the simultaneously played auditory target and distractor stimuli could either both belong to the same category (e.g., both non-flying, *cat - rat*), or two different ones (e.g., flying and non-flying, *duck - rat*). This is described as the *auditory congruence* (AC) with the levels *auditory congruent* and *auditory incongruent*.

##### 2.1.2.4 Visual congruence

**Figure 3:**
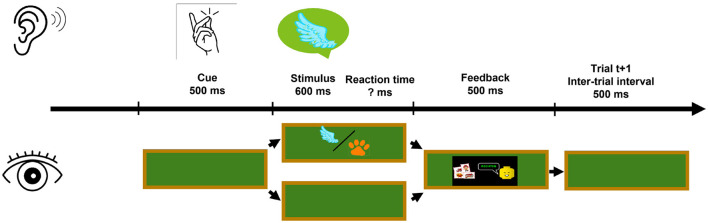
Depiction of one trial within the experiment. The trial structure for Experiment 1 is illustrated, where the visual stimulus was displayed on a virtual blackboard in half of the trials. When visible, the visual stimulus was played at the same time as the auditory stimulus. Its content could either be congruent or incongruent to the auditory target stimulus. Illustrated in the figure are only auditory target stimuli playing a flying animal name, indicated by the speech bubble containing a wing symbol.

Although the visual stimulus was irrelevant to the auditory task, it was hypothesized that its content would be processed. Thus, in addition to the auditory congruence, the content relation of the auditory target and the visual stimulus was classified as *visual congruence* (VC) with the levels *visually congruent* (e.g., *hearing cat - seeing a rat*) and *visually incongruent* (e.g., *hearing cat - seeing a duck*). In the example given in Figure 3, the auditory target stimulus represents a flying animal name, while the visual stimulus could present a flying or a non-flying animal.

##### 2.1.2.5 Visual display condition

In Experiment 1, the visual stimulus was displayed on the virtual blackboard in front of the participants in half of the trials. Thus, the visual stimulus was always spatially congruent with the frontal auditory stimulus. However, the task instruction was to focus on the auditory stimuli only. Thus, the visual stimulus was generally a distractor to the auditory stimuli. The visual display condition (VDC) had the levels *non-visible, visible-congruent*, and *visible-incongruent*. The latter two conditions are combinations of visibility and visual congruence. While the visibility was varied block-wise, the visual congruence was manipulated in each trial.

#### 2.1.3 Stimulus material

**Figure 4:**
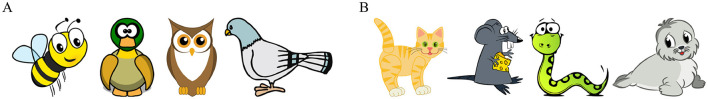
Visual stimuli. **(A)** Flying animals. From left to right: bee, duck, owl, dove. **(B)** Non-flying animals. From left to right: cat, rat, snake, seal.

In both experiments, auditory recordings of eight animal names in the German language were used. The animal names belonged to one of two categories, flying animals [“Biene” (bee), “Ente” (duck), “Eule” (owl), “Taube” (dove), Figure 4A], and non-flying animals [“Schlange” (snake), “Ratte” (rat), “Robbe” (seal), “Katze” (cat), Figure 4B]. Recordings were made in an anechoic chamber with a female adult (26 years) and a male child (5 years) using a Neumann TLM 170 microphone and a Zoom H6 recorder (70–20,000 Hz, 44.1 kHz sampling rate, 24-bit quantization). The acoustic stimuli were chosen based on a previous study by Loh et al. (2022) and are available in the ChildASA dataset by Loh and Fels (2023). The child and adult voices reflect daily teaching situations in German schools, where many teachers are female. Stimulus lengths were equalized to 600 ms using Audacity's “change tempo” algorithm (Audacity Team, 2022) As corresponding visual stimuli, royalty-free images of the animals were obtained from stock photo websites, namely Pixabay GmbH (2023), CleanPNG (2024), and PNGWing (2023), see also Figure 4.

The acoustic cue was the sound of snapping fingers played twice, which was obtained from Freesound (HeraclitoPD, 2024). The snapping was adjusted so the interval between the two snaps was 225 ms, which led to a total cue duration of 450 ms.

#### 2.1.4 Audio-visual reproduction

The auditory stimuli were reproduced using a dynamic binaural synthesis using the Virtual Acoustics auralization framework (Institute for Hearing Technology and Acoustics, RWTH Aachen University, 2021) version 2021 and the respective Unity package (Institute for Hearing Technology and Acoustics, RWTH Aachen University, 2023). A generic head-related transfer function (HRTF) of the IHTA artificial head with a resolution of 5° by 5° was used (Schmitz, 1995). This enabled spatial stimulus placement and accounted for participants' head movements. The stimuli were played back using Sennheiser HD 650 open headphones. To account for individual differences, perceptually robust headphone equalization filters (HpTF) (Masiero and Fels, 2011) were measured for each participant using the ITAtoolbox for Matlab (Dietrich et al., 2012). Sennheiser KE3 microphones were placed at the blocked ear canal entrance and recorded sweeps played back over the headphones. The measurement was repeated eight times, while re-adjusting the headphones between measurements. The resulting minimum-phase filter was calculated using an average of the measurements. Stimuli presentations at all four possible target and distractor positions were calibrated to 65 dB SPL.

**Figure 5:**
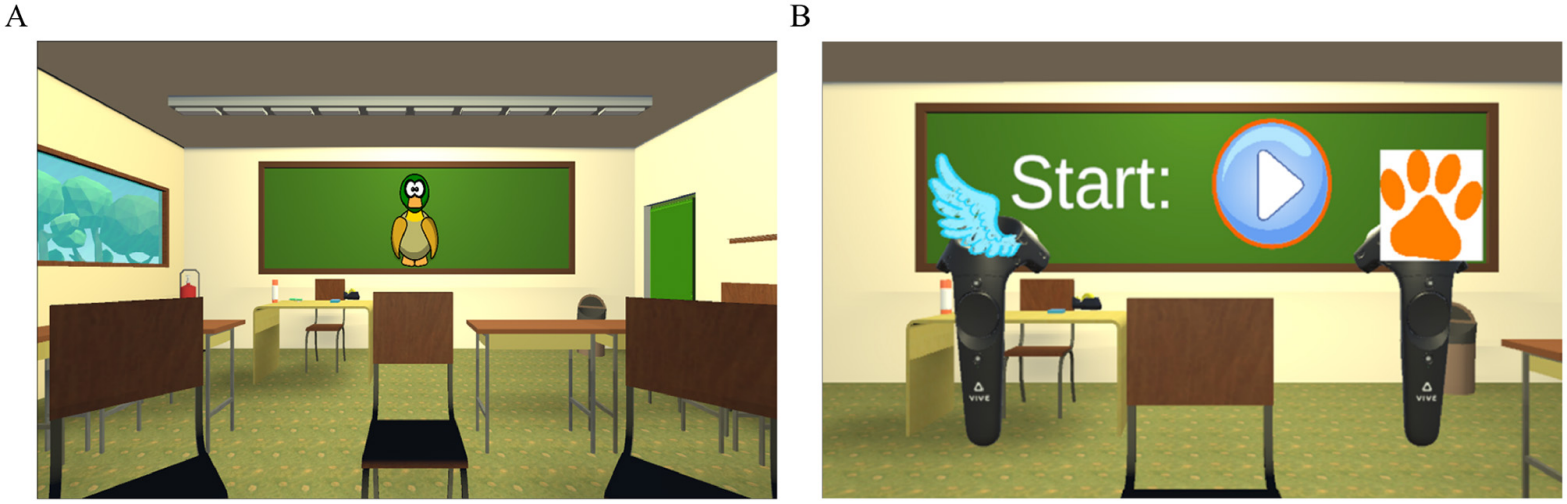
Virtual environment. **(A)** Virtual classroom with visual stimulus. **(B)** Virtual controllers.

For visual reproduction, a virtual classroom model was created using SketchUp make 2016 (Trimble Inc., 2022). The model was further refined in Unity 2019.4.21f (Unity Technologies, 2022), including furniture, an outdoor environment, and lighting. The virtual classroom measured (*l*×*w*×*h* = 10 × 9 × 3*m*^3^), larger than a typical classroom. Still, the paradigm design required a circle of chairs with a diameter of 2 m to represent the stimulus positions. The size of the classroom also allowed for a big blackboard to display all the instructions, feedback, and the visual stimulus (see Figure 5A). During the experiment, the participants faced the blackboard.

The Unity project, including the classroom model and experiment logic, as well as the Matlab code used to create the experiment configuration and HpTF measurement, is provided on Zenodo (Breuer et al., 2024).

The virtual classroom was presented using an HTC Vive Pro Eye head-mounted display (HMD). The participants were instructed to adjust the HMD to fit comfortably, and the vision was sharp. The interaction with the virtual world was accomplished using the HTC Vive controllers as depicted in Figure 5B. During the experiment, images of a paw and a wing reminded the participants which controller belonged to which response category. For a smooth visual reproduction of the virtual environment, a frame rate of 90 fps was obtained during the experiments.

#### 2.1.5 Experimental procedure

The overall experimental procedure was identical in both experiments. The structure of each trial is described in Figure 3. Each trial started with an inter-trial interval of 500 ms. This was followed by the auditory cue. The auditory stimuli were played back 500 ms after cue onset. The participants had unlimited time to respond and categorize the target stimulus. After each trial, feedback on whether the answer was correct was displayed for 500 ms, before the next inter-trial interval started.

In Experiment 1, an additional visual stimulus was displayed in front of the participants in half of the trials. The visual stimulus was shown concurrently with the auditory stimulus and remained visible until participants had responded, as depicted in Figure 3.

Both experiments included a training phase to get familiar with the task. During familiarization, participants looked around the virtual room to encourage head movements. Despite this, during the experiment, most participants looked to the front. The experiment was divided into ten experiment blocks with 72 trials each. Another feedback on the performance during the block was displayed after each block. Further, the progress of the whole experiment was displayed using a puzzle with an increasing number of visible parts at the end of each block. The whole experiment, including participant screening, lasted about 60 min.

The experiments were carried out at the Institute for Hearing Technology and Acoustics, RWTH Aachen University, Germany, in an acoustically treated hearing booth (*l*×*w*×*h* = 2.3 × 2.3 × 1.98*m*^3^), which offered a quiet environment. Before the experiment, informed consent was obtained from all participants, and an informal screening for German language skills, normal hearing, and vision was performed. If the participants passed, they could continue with the experiment. During the experiment, the participants were equipped with headphones, HMD, and the respective controllers to enter the virtual classroom.

#### 2.1.6 Hypotheses

Following prior studies (e.g., Koch et al., 2011; Fels et al., 2016; Loh et al., 2022; Breuer et al., 2022), several acoustic manipulations on ASA were included. Hypotheses H1–H3 are based on previous findings and build the foundation for this study, while the main incentive was to investigate cross-modal effects on the voluntary switching of auditory selective attention in a virtual classroom scenario (H4 and H5).

H1 Attention Transition: Switching attention increases error rates and response times (switch cost) due to the required re-orienting of the auditory attention to the target position.

H2 Target-Distractor Position-Combination: Task performance is expected to be highest in left-right, intermediate in next-to, and lowest in front-back conditions, reflecting the binaural reproduction and spatial distribution of the stimuli.

H3 Auditory Congruence: Auditory congruence improves performance relative to incongruence, because both acoustic stimuli yield the correct response.

H4 Visual Display Condition: The presence of visual stimuli is expected to increase response times due to additional processing.

H5 Visual Congruence: Cross-modal congruence enhances performance compared to incongruence due to the semantic processing of the stimulus material.

#### 2.1.7 Statistical analyses

The data were preprocessed according to a standard attention-/task-switching procedure and following previous studies using variations of the presented paradigm (e.g., Koch et al., 2011; Oberem et al., 2014, 2018; Loh et al., 2022; Breuer et al., 2022). Data were treated for implausibly short response times, because they were measured from the target stimulus onset, and extremely long ones. Error rates and response times were analyzed independently using generalized linear mixed effects models and checked for appropriate fits using residual diagnostics. Random effects were included based on theoretical considerations, which are outlined in the model descriptions below. Factor and interactions (fixed effects) were tested for statistical significance by performing parametric bootstrap likelihood ratio tests (McLachlan, 1987; Langeheine et al., 1996). Subsequent post-hoc tests were performed on model-estimated marginal means on the response scale, including bias-adjustments for random effects and residual variances when back-transformation of link scale estimates to the response scale involved non-linear transformations.

In detail, data preprocessing started with the exclusion of every first trial in each experiment block because the attention switch is uncontrolled for these trials (1.39%). Secondly, all trials following an erroneous response were excluded (15.77%). Lastly, trials with response times below 400 ms, or larger than the minimum of the individual 99% quantile and 6000 ms were removed (1.73%). To observe switching costs, that is, increased response times for successful task completion following an attention switch, all remaining error trials were removed for the response times analysis (11.75%). Error rates were analyzed on 81.11% of all trials, while correct response times were evaluated on 69.36% of the data.

Error rates were analyzed using a logistic mixed effects regression model (binomial response variable, logit link function) using the package glmmTMB 1.1.11 (Brooks et al., 2017) in R 4.3.3 (R Core Team, 2024). Models were fitted using maximum likelihood estimation with the default optimizer “nlminb”. The model formula was (*N*_*e*_, *N*_*c*_)~AT*AC*TD-PC*VDC+(1+*TD*-*PC* | *TpID*). Participants were included as a random effect for the intercept and target-distractor position-combination, accounting for individual performance differences and varying appropriateness of the reproduction HRTF, respectively.

Correct response times were transformed to response speeds *RS* = 400/*RT* and analyzed using a linear mixed effects model. Lognormal and Gamma models with log and identity links were considered (Lo and Andrews, 2015), however, residual diagnostics and data simulations suggested misfits. The model formula was 400/*RT*~AT*AC*TD-PC*VDC+(1 | *TpID*). Here, participants are only included as a random effect on the intercept, describing inter-individual differences in mean response speed.

Both models were checked for issues using the R package DHARMa 0.4.7 (Hartig, 2022), including the residual distribution, outliers, dispersion, homogeneity of variances, and zero- and one-inflation for the logistic regression model.

Statistical significance of factors and interactions was assessed using parametric bootstrap likelihood ratio tests (McLachlan, 1987; Langeheine et al., 1996) with 1,000 simulations for each comparison. Parametric bootstrap likelihood ratio tests compare two models, a null and an alternative model, which differ only by the factor (or interaction) under test. For the null model, the factor effect is enforced to be zero, while it can vary freely in the alternative model. An approximation of the likelihood ratio distribution under the null hypothesis, i.e., the factor has no effect, can be obtained by simulating responses from the null model and refitting both models to the simulated responses. By comparing the simulated likelihood ratio distribution with the observed likelihood ratio, it can be assessed whether the observed likelihood increase is expected under random variation in the data (null) or explained by an effect of the factor. The *p*-value is calculated as the proportion of simulated likelihood ratios that are larger than the observed one.

Subsequent post-hoc tests were performed on estimated marginal means using emmeans 1.11.1 (Lenth, 2025). All tests are performed on the response scale. Estimates on the response scale were obtained by estimating means and confidence intervals on the link scale and subsequently applying back-transformations for the link function (error rates model) or response transformation (response times model). These response-scale estimates include bias adjustments for variances modeled by random effects. Comparisons and statistics are reported after multiplicity adjustment using Holm's method. Tests for interactions were performed following simple contrasts, e.g., for the interaction of AC and TD-PC, pairwise comparisons for TD-PC would be performed for both levels of AC and vice versa.

All collected raw data, as well as the R code for the processing and evaluation, are provided on Zenodo (Breuer et al., 2024).

### 2.2 Results

**Figure 6:**
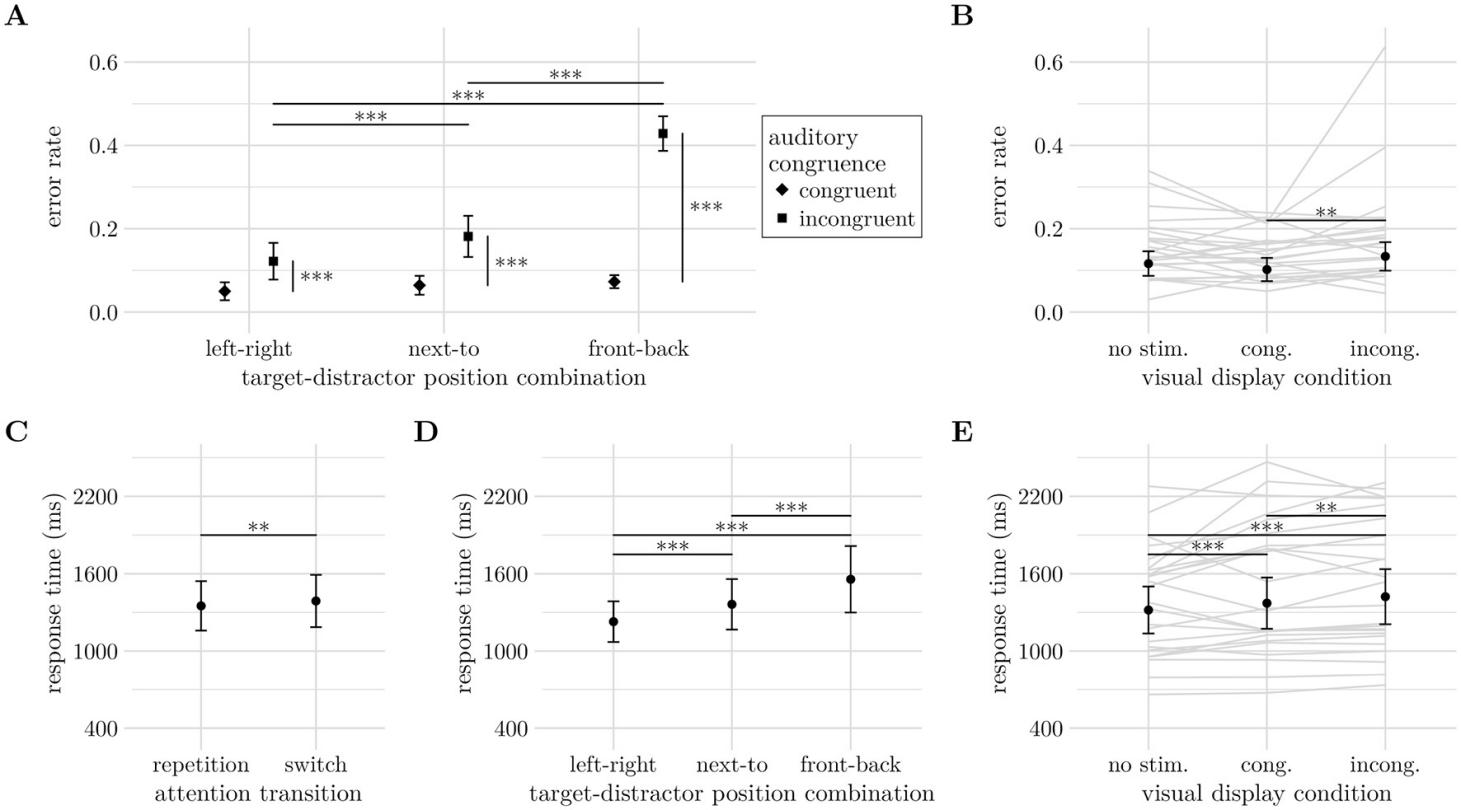
**(A)** The auditory congruence effect (AC, *M*_incong_−*M*_cong_) increases with spatially more difficult target-distractor position-combinations (TD-PCs). **(B)** Effect of visual display condition (VDC) on error rates. **(C)** Switching cost in correct response times. **(D)** More difficult spatial configurations of auditory target and distractor sources increased response times. **(E)** Presenting an animal picture with the auditory stimuli increased response times, particularly for incongruent pictures. Significance levels: * < 0.05, ** < 0.01, *** < 0.001. Error bars indicate unadjusted 95% confidence intervals. **(B, E)** Further show individual trend lines in the background.

The results of the parametric bootstrap likelihood ratio test are listed in Table 1 for both the error rates and response times analyses. Relevant effects are also plotted in Figure 6.

**Table 1:** Results of the parametric bootstrap likelihood ratio test with 1,000 simulations for error rates and response times.

**Predictor**		**Error rates**		**Response times**	
	*df*	−2Δ*LL*	*p*	−2Δ*LL*	*p*
**Main effects**					
AT	1	2.07	0.145	16.35	<**0.001**
AC	1	943.31	<**0.001**	0.45	0.531
TD-PC	2	37.97	<**0.001**	563.98	<**0.001**
VDC	2	11.51	**0.004**	63.56	<**0.001**
**Two-way interactions**					
AT x AC	1	0.95	0.365	0.02	0.895
AT x TD-PC	2	0.12	0.932	0.99	0.605
AC x TD-PC	2	100.27	<**0.001**	1.31	0.536
AT x VDC	2	0.83	0.663	3.49	0.180
AC x VDC	2	2.00	0.364	2.42	0.328
TD-PC x VDC	4	4.81	0.328	5.55	0.240
**Three-way interactions**					
AT x AC x TD-PC	2	0.49	0.783	3.01	0.238
AT x AC x VDC	2	2.14	0.355	2.23	0.349
AT x TD-PC x VDC	4	5.01	0.283	3.79	0.413
AC x TD-PC x VDC	4	2.39	0.681	0.67	0.953
**Four-way interaction**					
AT x AC x TD-PC x VDC	4	1.95	0.742	4.39	0.368

AT, attention transition; AC, auditory congruence; TD-PC, target-distractor position-combination; VDC, visual display condition and congruence. Significant effects are marked in bold.

In hypothesis (H1), we expected to observe a cost of the auditory attention switch in both error rates and response times. However, the parametric bootstrap likelihood ratio test only revealed a significant main effect of AT on response times (−2Δ*LL* = 16.35, *df* = 1, *p* < 0.001), but not error rates (−2Δ*LL* = 2.07, *df* = 1, *p* = 0.145). While not statistically significant, participants made slightly more errors on switch (*M* = 12.3%) than on repetition trials (*M* = 11%). When participants responded correctly, they were on average 38.1 ms (*CI* = [12.9, 63.3]*ms*) slower on switch trials than on repetition trials (*z* = 2.962, *p* = 0.003, c.f. Figure 6C).

Both the spatial positioning of the target and distractor stimuli (H2) and their congruence (H3) were expected to affect performance. Next to their main effects, we found a significant interaction effect between TD-PC and AC on error rates (−2Δ*LL* = 100.27, *df* = 2, *p* < 0.001, Figure 6A), while response times were only significantly influenced by TD-PC (−2Δ*LL* = 16.35, *df* = 1, *p* < 0.001, Figure 6D). With decreasing horizontal separation of target and distractor stimulus (left-right = 180°, next-to = 90°, front-back = 0°) correct responses became successively slower from the left-right configuration (*M* = 1, 228*ms*, *CI* = [1071, 1386]*ms*) to next-to (*M* = 1, 363*ms*, *CI* = [1167, 1558]*ms*, *z*_N − LR_ = 5.926, *p* < 0.001) and front-back (*M* = 1, 557*ms*, *CI* = [1299, 1815]*ms*, *z*_FB − N_ = 5.429, *p* < 0.001). The error rates showed an auditory congruence effect for all TD-PCs with higher error rates in incongruent trials (all Δ*M*≥7.22%, *z*≥4.967, *p* < 0.001). Comparing the size of the congruence effect across TD-PCs showed that it significantly increased from 7.22% (*CI*_LR_ = [4.37, 10.1]%) to 11.75% (*CI*_N_ = [8.42, 15.1]%) between left-right and next-to (*z* = 3.122, *p* = 0.0018), and from next-to to front-back at 35.55% (*CI*_FB_ = [32.02, 39.1]%, *z* = 13.676, *p* < 0.001). The increase of the auditory congruence effect was generated by a strong increase of error rates with decreasing horizontal separation on incongruent trials (all Δ*M*≥5.96%, *z*≥4.022, *p* < 0.001), while error rates on congruent trials increased slightly from left-right (*M* = 4.99%, *CI* = [2.83, 7.15]%) to front-back configurations (*M* = 7.29%, *CI* = [5.73, 8.84]%), *z* = 2.138, *p* = 0.0975.

Most important to the questions whether the visual stimulus is processed and affects performance in the attention switching task, we found a significant main effect of VDC on both error rates (−2Δ*LL* = 11.51, *df* = 2, *p* = 0.004, Figure 6A) and response times (−2Δ*LL* = 63.56, *df* = 2, *p* < 0.001, Figure 6E). Presenting the visual stimulus synchronously with the auditory stimuli increased response times from 1,318 ms (*CI* = [1136, 1501]*ms*) without a visual stimulus to 1,372 ms (*CI* = [1173, 1570]*ms*) for congruent (*z* = 3.526, *p* < 0.001) and 1,422 ms (*CI* = [1208, 1636]*ms*) for incongruent visual stimuli (*z* = 4.935, *p* < 0.001), respectively. Trials with incongruent visual stimuli also yielded slower response times than congruent ones (*z* = 2.800, *p* = 0.005). While the response times suggest a performance decrease consistent with (H4), the error rates show a different perspective that is in line with (H5). Error rates were lowest for visually congruent stimuli (*M* = 10.2%, *CI* = [7.41, 13]%), medium with no visual stimulus (*M* = 11.6%, *CI* = [8.68, 14.6]%) and highest with incongruent visual stimuli (*M* = 13.4%, *CI* = [9.94, 16.8]%). Performance in visually congruent trials was significantly better than in incongruent trials (*z* = 3.329, *p* = 0.003), while error rate differences with and without visual stimuli did not reach significance (*z*_vC − None_ = −1.876, *p* = 0.0620; *z*_vI − None_ = 2.157, *p* = 0.0620).

### 2.3 Discussion

Experiment 1 provides evidence that even task-irrelevant visual stimuli are processed during an auditory selective attention switching task. By extending the VR-based ASA task (Breuer et al., 2022) with concurrent visual stimuli, we show that previously reported ASA effects are modulated by their presence.

The observed switching cost (H1) replicates findings from earlier ASA research (Koch et al., 2011; Oberem et al., 2014; Breuer et al., 2022). The effect emerged only in response times, while error rates showed the same trend without reaching significance, consistent with prior work (Oberem et al., 2014, 2018; Loh et al., 2022; Breuer et al., 2022). This suggests that response times are more sensitive to attention switches than accuracy measures.

As expected, an interaction between target-distractor position-combination (H2) and auditory congruence (H3) emerged. In all TD-PC conditions, error rates were lower when target and distractor animals belonged to the same category, reflecting the main effect of AC. The difference was largest for front–back positions, indicating that horizontal separation of sources substantially affects task difficulty. This pattern may be amplified by the use of a generic HRTF, as front–back localization depends on individual monaural cues. Such confusions are a known limitation of binaural synthesis, but can be reduced using individualized HRTFs or real loudspeakers. Notably, Oberem et al. (2014) reported comparable effect sizes for generic and individual HRTFs, though the congruence benefit was slightly stronger for headphone-based reproduction.

Regarding hypotheses H4 and H5, both error rates and response times were influenced by the visual stimuli. Contrary to previous findings (Downing et al., 2015; Roberts et al., 2024), visual presentation increased response times and affected error rates depending on context (see Figures 6B, E). Importantly, in the current study, participants were instructed not to focus on the visual stimulus. Their ability to follow this instruction was supported by informal interviews conducted after the experiment, during which they were asked what they had looked at. Most participants reported glancing around the virtual room while generally maintaining a frontal view. To confirm this behavior, future work should include eye-tracking.

Individual data (Figure 6B) suggest participant-specific effects. While some showed higher error rates with incongruent visuals, others made fewer errors, reflecting typical inter-individual variability. This pattern may also indicate differences in how participants are influenced by the VR environment. Future work could explore individual susceptibility to virtual environments and its implications for audio-visual cognition, potentially informing personalized learning or gamified designs.

The visual stimulus in this task functioned as a task-irrelevant distractor rather than an additional target, which would be required for semantic priming (Barutchu et al., 2013). Nonetheless, consistent with multisensory enhancement (Stein and Stanford, 2008; Chng et al., 2019), congruent visuals supported correct responses, while incongruent ones impeded them. This indicates that even irrelevant visual input was integrated and influenced auditory attention.

Although response times and error rates showed opposing effects, both indicate that task-irrelevant visuals influenced auditory performance (Wegner-Clemens et al., 2024). Whether these effects reflect multisensory processing or strategic behavior remains unclear. Participants may have attempted to verify their auditory decision using the image when uncertain, explaining both longer reaction times and accuracy differences. If so, an interaction between VDC and TD-PC would be expected. Future studies should include post-experiment questionnaires to assess such strategies. Alternatively, the visual stimuli may have required additional semantic processing time, motivating Experiment 2, which tests whether a priming interval facilitates cross-modal integration.

## 3 Experiment 2: effects of visual priming on auditory selective attention

To test whether earlier semantic processing of the visual stimulus enhances the visual congruence effect, we conducted a second experiment on visual semantic priming. Here, the *stimulus onset asynchrony* (SOA) between visual and auditory stimuli was systematically varied.

### 3.1 Methods

The same stimulus material, audio-visual setup, and general procedure were used as in Experiment 1. The key difference was that the visual stimulus appeared in every trial, and its onset relative to the auditory stimulus was varied.

#### 3.1.1 Participants

Twenty-four new adults participated (age: 20–39 years, M = 25.08 years, SD = 4.13 years, 7 female). The same inclusion criteria and ethical statement applied as for Experiment 1.

#### 3.1.2 Experimental task and design

**Figure 7:**
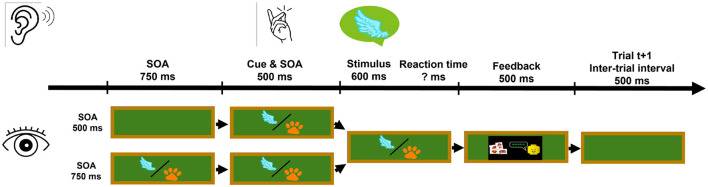
Depiction of one trial within Experiment 2, where the visual stimulus was displayed during all trials. While the auditory stimulus is still played at the same time as in Experiment 1, the visual stimulus is displayed before the auditory stimulus. Here, the stimulus onset was varied for SOA 500 ms or 750 ms.

The task and overall design were identical to Experiment 1 (see Figure 7). In Experiment 2, the visual stimulus was presented in every trial with an SOA of either 500 ms or 750 ms before the auditory stimuli. The factors Attention Transition, Target–Distractor Position–Combination, Auditory Congruence, and Visual Congruence were extended by the additional factor Stimulus Onset Asynchrony.

Previous research suggests that brain activity related to object categorization begins around 135 ms for visual and 305 ms for auditory stimuli (Iamshchinina et al., 2022). Visual priming effects on auditory processing have been reported for SOAs ranging from 200 ms (Holcomb and Anderson, 1993) to 1,000 ms (Liu et al., 2012), and even up to 2,400 ms (McQueen and Huettig, 2014). In related work on auditory attention preparation, Strivens et al. (2024) and Nolden et al. (2019) found that cue–stimulus intervals longer than 1,200 ms did not enhance switching when switch trials were frequent, as in the present design. Based on these findings and on timing requirements for audio–visual categorization (Breuer et al., 2022), we selected priming intervals of 500 ms and 750 ms. This allowed the visual stimulus to appear either simultaneously with or shortly before the auditory cue, while maintaining the overall trial duration from Experiment 1.

##### 3.1.2.1 Stimulus onset asynchrony

In Experiment 1, the visual stimulus appeared simultaneously with the auditory stimulus. In Experiment 2, two SOA levels were introduced: SOA750 and SOA500 (Figure 7). In the SOA750 condition, the image appeared 250 ms before the auditory cue and 750 ms before the auditory stimuli. In the SOA500 condition, it appeared simultaneously with the cue and 500 ms before the auditory stimuli. Each SOA was presented in half of the trials, balanced across participants and blocks.

#### 3.1.3 Hypotheses

The base hypotheses (H1–H3) from Experiment 1 also apply here.

H6 Visual Priming: Visual priming was expected to enhance processing of the visual stimulus, producing an audio–visual priming effect (H6). This should manifest as a stronger audio–visual congruence effect (as in H5 from Experiment 1). The effect was predicted to be larger in the SOA750 condition than in SOA500, since the visual stimulus could be processed before the auditory attention shift.

#### 3.1.4 Statistical analysis

Statistical analysis followed the same steps as in Experiment 1. Preprocessing procedures were identical, and model formulas were adjusted for the additional factor SOA. The error-rate model was (*N*_*e*_, *N*_*c*_)~AT*AC*TD-PC*SOA*VC+(1+*TD*-*PC* | *TpID*). The response-time model was 400/*RT*~AT*AC*TD-PC*SOA*VC+(1 | *TpID*).

A total of 16.56% of trials were excluded during preprocessing: 1.39% were first-in-block trials, 14.02% post-error trials, and 1.15% outliers from response-time processing. As in Experiment 1, only correct trials were analyzed for response times. Accordingly, error rates were based on 83.44% of all trials, and response times on 72.36%.

### 3.2 Results

Table 2 shows the results of the parametric bootstrap likelihood ratio tests of the error rates and response times models for Experiment 2. As can bee seen from the table, the error rates model resulted in a more complex effects structure compared to Experiment 1, while the response times effects remain similar.

**Table 2:** Results of the parametric bootstrap likelihood ratio test with 1,000 simulations for error rates and response times.

**Predictor**		**Error rates**		**Response times**	
	*df*	−2Δ*LL*	*p*	−2Δ*LL*	*p*
**Main effects**					
AT	1	4.44	**0.032**	28.48	<**0.001**
AC	1	898.44	<**0.001**	8.35	**0.003**
TD-PC	2	57.64	<**0.001**	1088.90	<**0.001**
SOA	1	0.07	0.779	0.14	0.712
VC	1	1.82	0.164	1.71	0.196
**Two-way interactions**					
AT x AC	1	0.64	0.427	0.54	0.473
AT x TD-PC	2	1.19	0.533	1.09	0.586
AC x TD-PC	2	162.78	<**0.001**	19.38	<**0.001**
AT x SOA	1	0.03	0.859	0.48	0.513
AC x SOA	1	0.43	0.514	1.94	0.168
TD-PC x SOA	2	3.05	0.218	3.74	0.136
AT x VC	1	1.90	0.167	0.83	0.334
AC x VC	1	0.20	0.634	0.07	0.770
TD-PC x VC	2	0.64	0.714	0.80	0.664
SOA x VC	1	1.29	0.264	1.11	0.300
**Three-way interactions**					
AT x AC x TD-PC	2	0.71	0.733	1.89	0.375
AT x AC x SOA	1	2.81	0.095	0.00	0.971
AT x TD-PC x SOA	2	1.09	0.582	1.24	0.529
AC x TD-PC x SOA	2	0.85	0.636	1.42	0.504
AT x AC x VC	1	0.02	0.865	0.15	0.688
AT x TD-PC x VC	2	6.84	**0.039**	3.09	0.204
AC x TD-PC x VC	2	0.20	0.904	2.15	0.324
AT x SOA x VC	1	0.91	0.339	0.00	0.980
AC x SOA x VC	1	2.71	0.116	0.85	0.358
TD-PC x SOA x VC	2	2.17	0.339	0.27	0.875
**Four-way interactions**					
AT x AC x TD-PC x SOA	2	0.18	0.917	1.48	0.472
AT x AC x TD-PC x VC	2	9.95	**0.011**	3.08	0.220
AT x AC x SOA x VC	1	3.32	*0.077*	0.71	0.401
AT x TD-PC x SOA x VC	2	3.01	0.229	3.68	0.167
AC x TD-PC x SOA x VC	2	6.54	**0.042**	0.99	0.606
**Five-way interaction**					
AT x AC x TD-PC x SOA x VC	2	3.78	0.151	0.24	0.882

AT, attention transition; AC, auditory congruence; TD-PC, target-distractor position-combination; SOA, stimuus onset asynchrony; VC, visual congruence. Significant effects are marked in bold.

**Figure 8:**
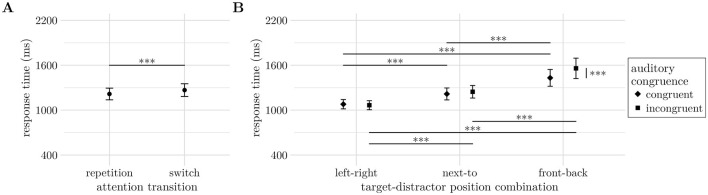
Experimental factors with statistically significant effects on response times. **(A)** Attention transition (AT). **(B)** Interaction of auditory congruence (AC) and target-distractor position-combination (TD-PC). Significance levels: * <0.05, ** <0.01, *** <0.001. Error bars indicate unadjusted 95% confidence intervals.

In contrast to Experiment 1, we found a main effect of attention transition on both error rates (−2Δ*LL* = 4.44, *df* = 1, *p* = 0.032) and response times (−2Δ*LL* = 28.48, *df* = 2, *p* < 0.001). For the error rates, however, AT is involved in significant higher level interactions, the highest being the interaction of attention transition, auditory congruence, target-distractor position-combination and visual congruence (−2Δ*LL* = 9.95, *df* = 2, *p* = 0.011). The response times showed the expect switching cost reflected by slower responses on switch trials (*M* = 1268*ms*, *CI* = [1, 183 1, 353]*ms*) compared to repetition trials (*M* = 1, 216*ms*, *CI* = [1, 138 1, 294]*ms*), *z* = 5.082, *p* < 0.001, see Figure 8A.

Another difference to Experiment 1 in response time effects is the presence of a main effect of auditory congruence (−2Δ*LL* = 8.35, *df* = 2, *p* = 0.003) and an interaction between auditory congruence and target-distractor position-combination (−2Δ*LL* = 19.38, *df* = 2, *p* < 0.001), see Figure 8B. The interaction occurs due to the emergence of an auditory congruence effect with decreasing horizontal separation of auditory target and distractor stimuli. At the left-right spatial configuration, response times did not differ between auditory congruent (*M* = 1, 079*ms*, *CI* = [1, 017 1, 141]*ms*) and incongruent trials (*M* = 1, 066*ms*, *CI* = [1, 005 1, 126]*ms*), *z* = −1.185, *p* = 0.236. For the front-back configuration, correct responses in incongruent trials (*M* = 1, 559*ms*, *CI* = [1, 422 1, 695]*ms*) were significantly slower than in congruent trials (*M* = 1, 431*ms*, *CI* = [1, 319 1, 543]*ms*), *z* = 4.331, *p* < 0.001.

**Figure 9:**
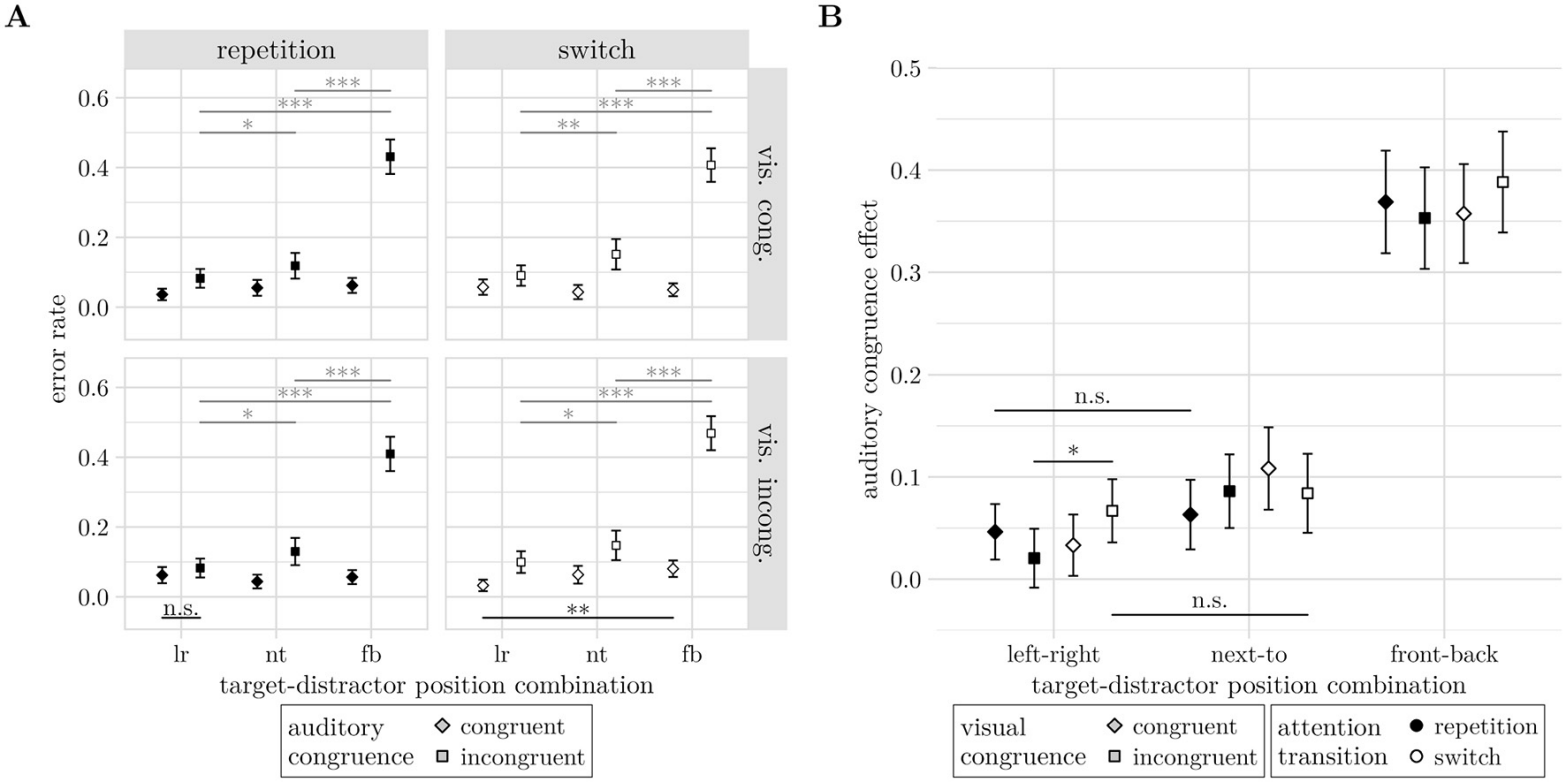
**(A)** Four-way interaction effect of attention transition, auditory congruence, target-distractor position-combination, and visual congruence (AT, AC, TD-PC, and VC) on error rates. Panels depict the sub-interaction of auditory congruence and target-distractor position-combination for the four factor combinations of attention transition and visual congruence. **(B)** Modulation of the auditory congruence effect (*M*_incong_−*M*_cong_) by visual congruence (shapes) and attention transition (fill color). In both **(A, B)**, significant differences across TD-PCs are not annotated for readability, instead, non-significant (n.s.) contrasts are shown. Significance levels: * <0.05, ** <0.01, *** <0.001. Error bars indicate unadjusted 95% confidence intervals.

The four-way interaction of AT, AC, TD-PC, and VC was significant (−2Δ*LL* = 9.95, *df* = 2, *p* = 0.011), see Figure 9. In almost all factor combinations of this interaction, the auditory congruence effect resulted in a significant increase of errors by at least 3.32% (all *z*≥2.167, *p* ≤ 0.030). Only for Repetition trials in the left-right configuration and an incongruent visual stimulus did the auditory congruence effect not reach significance, although it showed the expected error rate increase (*M* = 2.04%, *CI* = [−0.84 4.93]%, *z* = 1.389, *p* = 0.165).

Comparing the auditory congruence effect across TD-PCs resulted in a diagonal pattern of non-significant increases, see Figure 9B. The auditory congruence effect increased with smaller horizontal separation of target and distractor for switch trials with congruent visual stimuli (all Δ*M*≥7.5%, *z*≥3.08, *p* ≤ 0.002), and repetition trials with incongruent visual stimuli (all Δ*M*≥6.55%, *z*≥2.87, *p* ≤ 0.004). In contrast, the auditory congruence effect did not increase from left-right to next-to configurations for switch trials with incongruent visual stimuli (Δ*M* = 1.72%, *z* = 0.74, *p* = 0.459), and for repetition trials with congruent visual stimuli (Δ*M* = 1.69%, *z* = 0.806, *p* = 0.420).

To test whether the cross pattern arises from interactions of the auditory congruence effect with attention transition or visual congruence, we compared the size of the auditory congruence effect between switch and repetition trials for all factor combinations of TD-PC and VC, and between visually congruent and incongruent trials for all factor combinations of AT and TD-PC. The latter comparisons, testing the interaction of auditory and visual congruence, did not reveal significantly different auditory congruence effects between visually congruent and incongruent trials (all |Δ*M*| ≤ 3.36%, |*z*| ≤ 1.595, *p*≥0.111). Note, though, that the largest size of the visual congruence effect observed here is similar to that found in Experiment 1. However, as illustrated in Figure 9B, testing the interaction of auditory congruence with attention transition showed a larger auditory congruence effect in Switch compared to repetition trials when the target-distractor configuration was left-right and the visual stimulus was incongruent (*M* = 4.64%, *CI* = [0.53 8.75]%, *z* = 2.21, *p* = 0.027). The auditory congruence effect also tended to be larger on switch compared to repetition trials in the next-to configuration with congruent visual stimuli, although the difference did not reach significance (*M* = 4.51%, *CI* = [−0.23 9.25]%, *z* = 1.87, *p* = 0.062).

**Figure 10:**
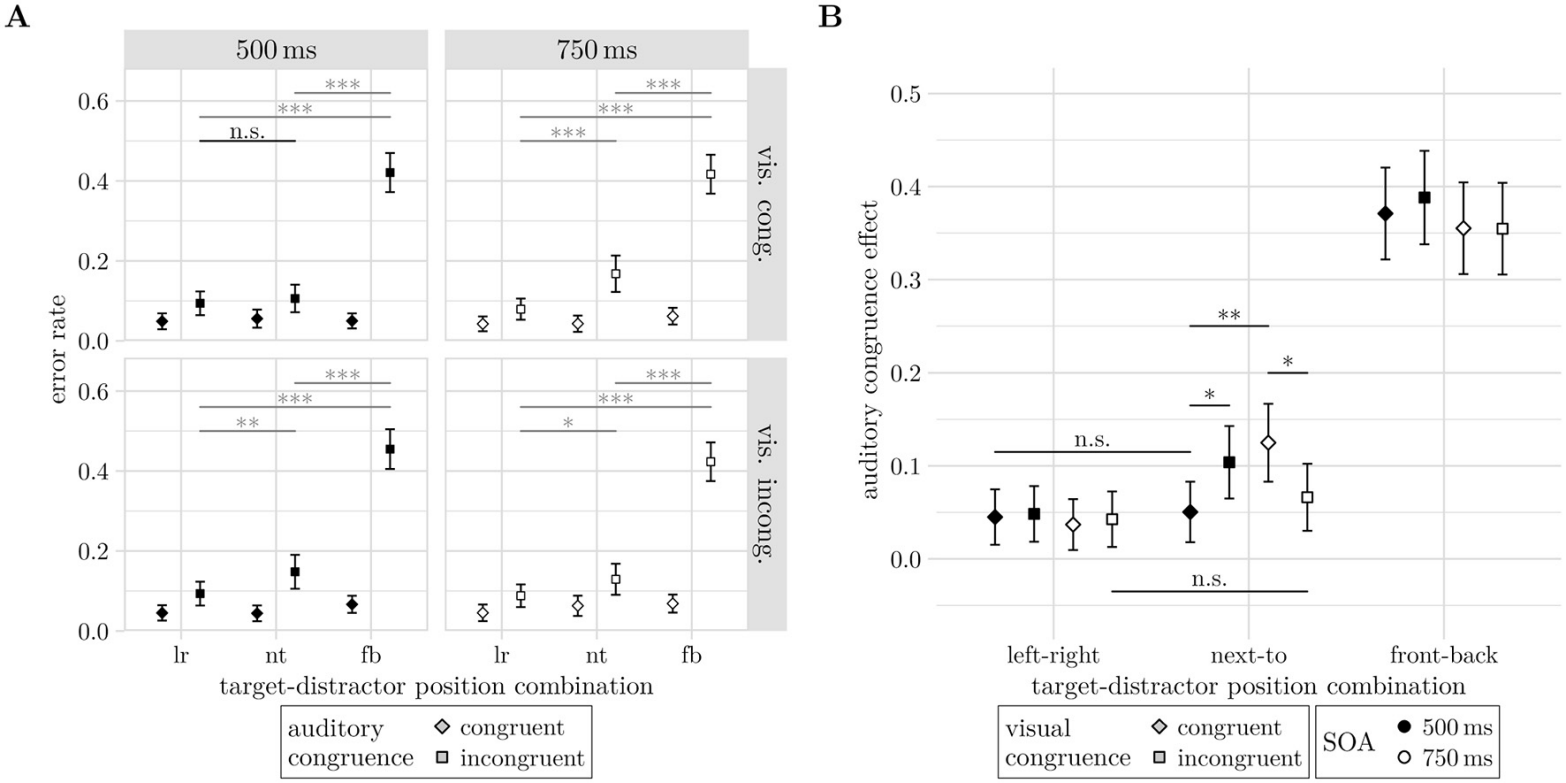
**(A)** Four way interaction effect of auditory congruence, target-distractor position-combination, stimulus onset asynchrony, and visual congruence (AC, TD-PC, SOA, and VC) on error rates. Panels depict the sub-interaction of auditory congruence and target-distractor position-combination for the four factor combinations of stimulus onset asynchrony and visual congruence. **(B)** Modulation of the auditory congruence effect (*M*_incong_−*M*_cong_) by visual congruence (shapes) and stimulus onset asynchrony (fill color). In both **(A, B)**, significant differences across target-distractor position-combinations are not annotated for readability, instead non-significant (n.s.) contrasts are shown. Significance levels: * <0.05, ** <0.01, *** <0.001. Error bars indicate unadjusted 95% confidence intervals.

Also, the four-way interaction of AC, TD-PC, SOA and VC was significant (−2Δ*LL* = 6.54, *df* = 2, *p* = 0.042), see Figure 10. All factor combinations of TD-PC, SOA, and VC showed a significant auditory congruence effect (all Δ*M*≥3.69%, *z*≥2.65, *p* ≤ 0.008). Comparing the auditory congruence effects across TD-PC levels for all combinations of SOA and VC suggested a second cross-pattern. Comparisons of the auditory congruence effect between left-right and next-to configurations revealed significant increases for congruent visual stimuli presented with 750 ms SOA (Δ*M* = 8.81%, *CI* = [3.10 14.51]%, *z* = 3.70, *p* < 0.001) and for incongruent visual stimuli presented at 500 ms SOA (Δ*M* = 5.55%, *CI* = [ <0.01 11.1]%, *z* = 2.39, *p* = 0.017). Conversely, the same comparisons were not significant for congruent visual stimuli presented with 500 ms SOA (Δ*M* = 0.55%, *CI* = [−4.62 5.71]%, *z* = 0.25, *p* = 0.799) and for incongruent visual stimuli presented at 750 ms SOA (Δ*M* = 2.36%, *CI* = [−3.1 7.81]%, *z* = 1.03, *p* = 0.301).

Testing the auditory congruence effect for interactions with SOA and visual congruence (see Figure 10B), reveals that both had a TD-PC dependent effect on the error rates. In the next-to condition, contrasts between incongruent and congruent visual stimuli showed a larger auditory congruence effect for incongruent visual stimuli when they were presented at 500 ms SOA (*M* = 5.34%, *CI* = [0.70 0.98]%, *z* = 2.26, *p* = 0.024). Surprisingly, the auditory congruence effect was lower for incongruent relative to congruent visual stimuli at 750 ms SOA (*M* = −5.87%, *CI* = [−10.80 −0.94]%, *z* = −2.33, *p* = 0.020). Other spatial configurations of target and distractor were not significantly affected by visual congruence (all |*z*| ≤ 0.53, *p*≥0.59).

Lastly, the auditory congruence effect decreased from 750 ms to 500 ms SOA only for congruent visual stimuli and the next-to target-distractor configuration (Δ*M* = −7.45%, *CI* = [−12.27 −2.63]%, *z* = −3.03, *p* = 0.002). None of the other factor combinations of TD-PC and VC showed significant changes of the auditory congruence effect between SOA levels (all |Δ*M*| ≤ 3.76, |*z*| ≤ 1.54, *p*≥0.123).

### 3.3 Discussion

Building on the findings of Experiment 1, Experiment 2 examined whether providing additional time for visual processing would facilitate cross-modal integration and audio-visual congruence effects. In Experiment 2, the voluntary ASA switching task in VR was extended by introducing a visual prime preceding the auditory target stimulus to investigate whether a priming interval would facilitate cross-modal integration and audio-visual congruence effects.

Consistent with previous studies (e.g., Koch et al., 2011; Fels et al., 2016; Oberem et al., 2014; Breuer et al., 2022), as well as Experiment 1, attention transition, auditory congruence, and target-distractor position-combination (hypotheses H1–H3) were found to affect correct response times (see Figure 8). Since these effects have already been discussed in Experiment 1, we do not elaborate on these findings further.

Despite these expected benefits, the results revealed no overall audio-visual priming advantage in terms of reduced response times or error rates for congruent prime–target pairs compared to incongruent pairs (H5) or a clear modulation with the stimulus onset asynchrony (H6). This finding stands in contrast to previous laboratory studies (Holcomb and Anderson, 1993; Kircher et al., 2009; McQueen and Huettig, 2014) reporting semantic priming benefits. However, those studies typically employed lexical decision tasks without competing distractors, whereas in our paradigm, participants had to perform an auditory selective attention task with simultaneous distractor sounds from other spatial locations.

Although we only hypothesized audio-visual congruence effects (H5, H6) for Experiment 2, we found interactions involving attention transition. Without specific hypotheses, task difficulty can still be expected to increase from repetition to switching of attention, from auditory and visually congruent to incongruent stimuli, and from left-right over next-to to front-back spatial configurations. From this perspective, the absence of a growing auditory congruence effect under visually congruent conditions likely reflects a floor effect, where performance approached ceiling accuracy, leaving little room for improvement. The same interpretation holds for the insignificant auditory congruence effect for visually incongruent stimuli in repetition trials with the left-right condition. On the other hand, the congruent visual stimulus was able to facilitate the auditory decision in repetition trials, which resulted in a similar auditory congruence effect for left-right and next-to conditions. Thus, the visual stimulus helped overcome the more difficult spatial discrimination in the next-to condition. Note that with 15 repetitions per condition, it would take, on average, one less error trial per participant to decrease the error rate by 6.67%. Though this estimate does not consider preprocessing and modeling influences, it serves to demonstrate the small size of the observed differences.

The main result from the four-way interaction of auditory congruence, target-distractor position-combination, visual congruence and stimulus onset asynchrony (see Figure 10) was that congruent visual stimuli minimized the auditory congruence effect between left-right and next-to conditions in SOA 500 ms trials, while incongruent visual stimuli decreased the auditory congruence effect in the next-to condition for SOA 750 ms trials to reach left-right trial performance. As illustrated in Figure 10A by non-significance markers, a significantly higher auditory congruence effect was observed between 500 ms and 750 ms in next-to conditions for visually congruent cases. Further, only in next-to cases and the SOA 500 ms condition, visually incongruent stimuli influenced auditory congruence more than congruent stimuli. The opposite effect was found for the SOA 750 ms condition. In combination, it seems that the visual stimulus supported auditory decision making less with longer presentation, indicating a semantic priming inhibition of return effect, i.e., a reduced facilitation when the prime–target interval becomes too long (Weger and Inhoff, 2006). This suggests that while the visual stimulus was processed and influenced auditory decision making, the strength of this effect can indeed be modulated by presentation time, as is expected from visual priming (McQueen and Huettig, 2014; Liu et al., 2012; Kircher et al., 2009).

Nonetheless, the results of both four-way interactions are challenging to interpret and seem to be inconsistent in parts. Thus, while there is a cross-modal effect introduced by the visual stimulus and modulated by the stimulus onset asynchrony, these results need to be taken with caution and should be validated in follow-up investigations. One approach could be to investigate even longer priming intervals. Further, future investigations should consider adapting the acoustic playback or spatial distribution of sound sources to overcome the possible floor and ceiling effects in the TD-PC left-right and front-back conditions. One approach could be shifting the setup by 45° in the horizontal plane to avoid acoustically extreme positions.

Despite the limited evidence, Experiment 2 demonstrates that even task-irrelevant visual stimuli can modulate auditory decision-making when presented within specific temporal windows. These insights contribute to understanding how temporal alignment affects multisensory attention in immersive environments.

## 4 General discussion

The presented studies examined how visual stimuli influence auditory selective attention switching using a child-appropriate paradigm adapted from Loh et al. (2022) in a virtual classroom scenario (see also Breuer et al., 2022). Experiment 1 investigated cross-modal integration of task-irrelevant visual stimuli, whereas Experiment 2 examined whether varying the stimulus onset asynchrony (SOA) would enhance visual processing and facilitate audio-visual integration.

The auditory effects reported in previous studies (Koch et al., 2011; Oberem et al., 2014; Breuer et al., 2022) were successfully replicated in both experiments. Consistent with the hypothesis that reorienting auditory attention entails switching costs (H1), we observed increased error rates and prolonged response times in switch trials compared to repetition trials. Moreover, greater spatial separation between target and distractor sounds reduced task difficulty (H2). Finally, an auditory congruence effect (H3) emerged, with better performance in congruent relative to incongruent trials.

In Experiment 1, a semantic priming effect was introduced by the task-irrelevant visual stimulus, evident in both error rates and reaction times. This finding aligns with previous literature on multisensory integration (e.g., Stein and Stanford, 2008) and indicates that even task-irrelevant visual information was processed. Performance declined in visually incongruent trials, further supporting the integration of the visual distractor (see, e.g., Nah et al., 2021).

Experiment 2 investigated whether allowing more time for visual processing could enhance audio-visual interactions. The visual stimulus was presented either 500 ms or 750 ms before the auditory target onset. Two four-way interactions indicated an audio-visual congruence effect modulated by both the stimulus-onset asynchrony and the auditory task difficulty. Although the visual stimulus was processed, the resulting audio-visual congruence effects remained comparatively small.

While previous studies such as Holcomb and Anderson (1993) and McQueen and Huettig (2014) found that longer exposure to the visual stimuli facilitates the cross-modal evaluation of the audio-visual stimuli, the present results show a different trend. Here, prolonged prime presentation times may have reduced priming effectiveness. As the visual stimulus did not predict the correct response, manipulating the ratio of visually congruent to incongruent trials might encourage stronger engagement with the visual stimulus (see Strivens et al., 2024). Another way to enhance cross-modal integration could be to show an identical visual stimulus to the auditory target. Furthermore, the investigated SOAs might have been too long to measure larger effects of the visual stimuli, given that visual object categorization starts from 135 ms (Iamshchinina et al., 2022).

The SOA-mediated effect of the visual stimulus on the auditory congruence effect suggests that participants attempted to process the visual stimulus during the interval between cue and target–distractor presentation. However, it seems counterintuitive that the effect should change direction from a 500 ms to a 750 ms SOA, unless a semantic inhibition of return effect is assumed. Weger and Inhoff (2006) argue that inhibition of return for semantic and spatial dimensions is independent, yet both may have contributed to the observed pattern.

Another possible explanation concerns the overall task difficulty. Strivens et al. (2024) noted that the adult version of this paradigm (Koch et al., 2011) may be relatively easy, limiting observable effects. To increase task difficulty and elicit stronger switch costs, future work could manipulate the frequency of attention switches or the proportion of incongruent auditory trials. Similarly, other studies using variants of this paradigm (Breuer et al., 2022; Loh et al., 2022; Fels et al., 2016) have reported low overall error rates, suggesting that difficulty could be increased by adding background noise or increasing the number of repetitions per condition. yet with the trade-off of longer sessions and potential participant fatigue.

Regarding the aim of creating more plausible research scenarios using VR, one drawback in the current study design is the interaction with the VR environment. Participants in the current VR study moved their heads only rarely, despite being encouraged to do so during training. Since the instructions and feedback were displayed in front of them, their focus remained largely fixed. This behavior was also observed by Breuer et al. (2022). In the present design, the visual distractor always appeared from the front, which may have limited cross-modal effects. According to the spatial rule, cross-modal stimuli presented from the same location show the strongest integration (Spence, 2013). Future studies could therefore test whether spatially distributed visual stimuli enhance semantic priming and further examine how such manipulations affect immersion and task plausibility in VR.

Consistent with calls for more realistic research environments (e.g., Weger and Inhoff, 2006), the present study advances this direction by demonstrating how complex cross-modal interactions can be examined in closer-to-real-life VR contexts. While the broader influence of VR on auditory selective attention and related cognitive mechanisms remains underexplored (Albus and Seufert, 2023; Wenk et al., 2023), the current work contributes to this area by replicating key effects from both VR-based (Breuer et al., 2022) and audio-only versions of the task (Loh et al., 2022; Oberem et al., 2014; Koch et al., 2011). The findings confirm that established ASA mechanisms persist in more immersive, audio-visual environments.

This supports the view that, although VR introduces greater plausibility and multimodal complexity, it remains a viable and reproducible platform for cognitive research. It enables the investigation of perception and attention under conditions that more closely resemble real-world challenges, such as those relevant to hearing aid development or everyday communication in noisy environments. However, the current study did not assess participants sense of immersion or the perceived quality of the virtual environment. Breuer et al. (2022) reported that established measures such as the Simulator Sickness Questionnaire (Kennedy et al., 1993) and the Igroup Presence Questionnaire (Schubert, 2003) were not suitable for this specific task and participant group, likely because these tools were designed for other applications. Future work should therefore develop or adapt appropriate measures to assess immersion and presence within this paradigm and explore their relation to cross-modal perception and auditory selective attention.

To establish a foundation for developmental research on cross-modal influences in auditory selective attention, this study employed a classroom environment and a child-appropriate task. Although Loh et al. (2022) validated the task for children aged 6–10, the suitability of consumer-grade VR hardware for young children remains uncertain. The HTC Vive Pro Eye headset used in this study has no explicit age restriction, but it was not designed for children (HTC Corporation, 2025). Other manufacturers recommend use only from ages 10–13 and older (Meta, 2025; Pico Technology Co. Ltd., 2025). Beyond content-related concerns, current VR hardware is not optimized for children's physiology. For instance, the interpupillary distance (IPD) calibrated for adults typically reaches stable values only around age 16 (MacLachlan and Howland, 2002). Nonetheless, both research and commercial tools have successfully employed VR with children as young as six years (Coleman et al., 2019; Cognitive Leap Solutions Inc., 2025; Giunti Psychometrics, 2025). Ethical considerations remain essential, including detailed participant and guardian information about potential risks such as cybersickness, headset discomfort, or difficulties distinguishing virtual from real environments (Kaimara et al., 2021).

In summary, the presented studies partially confirmed the cross-modal processing of task-irrelevant visual stimuli in an auditory selective attention task. Although participants were instructed to ignore the visual stimuli, behavioral effects, prolonged response times in Experiment 1, and four-way interactions in error rates in Experiment 2, indicate that visual information was processed. However, no conclusive audio-visual congruence effect was found. From the perspective of multimodal enhancement, audio-visually congruent stimuli were expected to improve performance, while incongruent stimuli would impair it. This leaves the question open to what extent the visual stimuli were processed semantically. The observed response time increases and selective visual congruence effects suggest that visual stimuli may have been used to verify auditory decisions rather than integrated automatically. Still, more research is required to understand the time course of cross-modal priming and the mechanisms that distinguish it from multisensory integration, which occurs within 300 ms of stimulus onset (Stevenson and Wallace, 2013).

In conclusion, the two experiments highlight that findings from controlled laboratory paradigms do not always translate directly to more naturalistic VR scenarios. This work provides an important step toward understanding how semantic and spatial factors interact in immersive contexts and emphasizes the importance of considering spatial attention demands when examining cross-modal effects in VR. It thereby contributes to bridging the gap between simplified multisensory research and real-world applications, particularly for VR-based learning and training scenarios.

## Data Availability

The datasets presented in this study can be found in online repositories. This data can be found here: https://zenodo.org/records/13380476.
